# Mst1 Knockout Alleviates Mitochondrial Fission and Mitigates Left Ventricular Remodeling in the Development of Diabetic Cardiomyopathy

**DOI:** 10.3389/fcell.2020.628842

**Published:** 2021-01-21

**Authors:** Xinyu Feng, Shanjie Wang, Xingjun Yang, Jie Lin, Wanrong Man, Yuan Dong, Yan Zhang, Zhijing Zhao, Haichang Wang, Dongdong Sun

**Affiliations:** ^1^Heart Hospital, Xi'an International Medical Center, Xi'an, China; ^2^Department of Cardiology, Xijing Hospital, Fourth Military Medical University, Xi'an, China

**Keywords:** Mammalian Sterile 20-like Kinase 1, Mst1, Mitochondrial Fission, Mitochondrial Fusion, Mitochondrial Dysfunction, Diabetic Cardiomyopathy, DCM

## Abstract

The disruption of mitochondrial dynamics is responsible for the development of diabetic cardiomyopathy (DCM). However, the mechanisms that regulate the balance of mitochondrial fission and fusion are not well-understood. Wild-type, Mst1 transgenic and Mst1 knockout mice were induced with experimental diabetes by streptozotocin injection. In addition, primary neonatal cardiomyocytes were isolated and cultured to simulate diabetes to explore the mechanisms. Echocardiograms and hemodynamic measurements revealed that Mst1 knockout alleviated left ventricular remodeling and cardiac dysfunction in diabetic mice. Mst1 knockdown significantly decreased the number of TUNEL-positive cardiomyocytes subjected to high-glucose (HG) medium culture. Immunofluorescence study indicated that Mst1 overexpression enhanced, while Mst1 knockdown mitigated mitochondrial fission in DCM. Mst1 participated in the regulation of mitochondrial fission by upregulating the expression of Drp1, activating Drp1^S616^ phosphorylation and Drp1^S637^ dephosphorylation, as well as promoting Drp1 recruitment to the mitochondria. Furthermore, Drp1 knockdown abolished the effects of Mst1 on mitochondrial fission, mitochondrial membrane potential and mitochondrial dysfunction in cardiomyocytes subjected to HG treatment. These results indicated that Mst1 knockout inhibits mitochondrial fission and alleviates left ventricular remodeling thus prevents the development of DCM.

## Introduction

The International Diabetes Federation (IDF) highlights that the global prevalence of diabetes has been increasing over recent decades. The prevalence of diabetes was estimated to be 8.8% in 2015 and is predicted to rise to 10.4% in 2040 (Ogurtsova et al., 2017). Diabetes itself is an independent risk factor for cardiovascular disease, and the increased prevalence of diabetes has led to more cases of cardiovascular complications (Adeva-Andany et al., 2019). With the chronic and progressive damage of diabetes, diabetic cardiomyopathy (DCM) which is characterized by early cardiac diastolic dysfunction, decreased or preserved systolic function and a reduced ejection fraction eventually resulting in heart failure, can be caused (Rubler et al., 1972). It is important to elucidate the mechanism of diabetic cardiomyopathy to reduce the cardiac mortality of diabetic patients.

A universal theory exists across science fields that mitochondria are not static, solitary organelles, but that they constantly undergo shape and number changes due to the mitochondrial dynamics (Williams and Caino, 2018). Mitochondrial dynamics are mainly controlled by the two opposing processes of fission and fusion (Mattie et al., 2019). Dynamin-related protein 1 (DRP1), mitochondrial fission 1 (Fis1), mitochondrial fission factor (MFF) and mitochondrial dynamics proteins (MiD49/MiD51) constitute the core machinery promoting mitochondrial fusion. Mitofusin 1/2 (MFN1/2) and optic atrophy protein 1 (OPA1) achieve fusion of the outer and inner mitochondrial membranes, respectively (van der Bliek et al., 2013). Numerous experiments have observed that hyperglycemia induces mitochondrial fragmentation (Yu et al., 2006, 2011). Dysregulation of mitochondrial dynamics has been hypothesized to contribute to the pathogenic progression of metabolic diseases, including the diabetic complication of DCM (Galloway and Yoon, 2015). However, the molecular mechanisms responsible for mitochondrial dynamics in diabetic stress are not well-understood.

Mammalian sterile 20-like kinase 1 (Mst1) is the core component of the Hippo pathway. It is expressed in various organs and tissues of mammals, especially in cardiomyocytes. The Hippo pathway associated with Mst1 regulates the growth and death of myocardial cells (Yang et al., 2018). We have discussed the role of Mst1 in regulating autophagy and apoptosis in the cardiovascular system, as well as the cardiac function of Mst1 in diabetic mice (Zhang et al., 2016; Wang et al., 2018a). Our previous study demonstrated that the expression of Mst1 increased DCM progression, while other studies showed that mitochondrial fission is increased during DCM progression. Therefore, the purpose of this study was to determine the regulatory effect of Mst1 on mitochondrial fission and fusion of the myocardium under hyperglycemic conditions.

## Materials and Methods

### Animals and Treatment

Mst1 transgenic (Mst1Tg) and Mst1 knockout (Mst1^−/−^) mice (C57BL/6 background) were purchased from K&D gene technology (Wu Han, China). All animals were identified by Western blot and real-time PCR analysis. The present study was performed according to the NIH guidelines on the use of laboratory animals. All protocols were approved by the Institutional Animal Care in the Xi'an International Medical Center. As previously described (Zhang et al., 2016), 8-week old mice (Male, 20–25 g) were intraperitoneally injected with streptozotocin (STZ, 50 mg/kg, dissolved in 100 mmol/L citrate buffers, pH 4.5) for 5 days and fed a high-fat and high-sugar diet to induce a diabetes model, and only those with random blood glucose levels ≥16.6 mmol/L twice were labeled having diabetes. Four groups were set as follows: (1) Wild type group (WT, *n* = 8); (2) Diabetes group (DM, *n* = 10); (3) Diabetes + Mst1^−/−^ group (DM+Mst1^−/−^, *n* = 11); (4) Diabetes + Mst1Tg group (DM+Mst1Tg, *n* = 9).

### Isolation of Primary Neonatal Mouse Cardiomyocytes

Isolation of primary neonatal mouse cardiomyocytes was described previously (Ehler et al., 2013). Briefly, neonatal mouse hearts were quickly excised and minced into fragments prior to enzymatic digestion with a collagenase/dispase mixture (Invitrogen, USA). After several rounds of digestion, the digested fragments were placed on a sterilized platform to sediment for several minutes, and digested cells in supernatants were pre-placed for 90 min to remove fibroblasts and endothelial cells because of their earlier attachment. Then the residual supernatant with abundant cardiomyocytes was replanted in collagen-coated dishes at ~1.5 × 10^5^ cells per cm^2^. After incubation without being moved for 48 h, the culture medium was refreshed with complete medium.

### The Culture of Primary Neonatal Mouse Cardiomyocytes

The culture of primary cardiomyocytes was described previously (Ehler et al., 2013). Briefly, cells were cultured in complete Dulbecco's Modified Eagle's Medium (DMEM, HyClone, USA) with 4,500 mg/L glucose,4 mM L-glutamine, 110 mg/L sodium pyruvate, 1% (v/v) penicillin/streptomycin and 10% (v/v) fetal bovine serum (FBS, Biological Industries). Cells were then placed in an incubator containing 95% air and 5% CO_2_ at 37°C. The culture medium was replaced every 2–3 days. For the *in vitro* study, primary cardiomyocytes were treated with low glucose (LG, 5.5 mM/L) and high glucose (HG, 30 mM/L) for 48 h.

### Cardiac Function Evaluation

After a three-month duration of the diabetic condition, all mice were carefully anesthetized with 2% isoflurane and fixed on a heating pad (37°C) in a supine position. Cardiac function was evaluated across the thoracic region using 2-D Guided M-mode echocardiography (VisualSonics Vevo 2100, Toronto, ON, Canada) equipped with a 15 MHz linear transducer. Left ventricular end-diastolic diameter (LVEDD) and left ventricular end-systolic diameter (LVESD) were measured on the left ventricular short axis. Left ventricular ejection fraction (LVEF) and left ventricular fraction shortening (LVFS) were calculated by computer algorithms, as described previously. A pressure catheter was used to assess the maximal rate of LV contractility and relaxation with a previously described method (Wang et al., 2018b).

### Cell Apoptosis Assay

A terminal deoxynucleotidyl transferased UTP nick end labeling (TUNEL) assay kit (Roche Applied Science, Swiss) was employed to determine myocardial apoptosis following the manufacturer's instructions as previously described (Zhang et al., 2016).

### Transmission Electron Microscopy (TEM)

Murine heart tissues were fixed in 2% glutaraldehyde for at least 24 h. Tissues were then immersed in 2% osmium tetroxide and 1% aqueous uranyl acetate, each for 1 h. After being washed with a series of ethanol solutions (50, 70, 90, and 100%), tissues were transferred to propylene oxide, incubated in a 1:1 mixture of propylene oxide and EMbed 812 (Electron Microscopy Sciences) for 1 h and then placed in a 70°C oven to polymerize. Sections (75–80 nm) were cut using a Leica ultramicrotome equipped with a Diatome diamond knife and collected on 200-mesh copper grids. After being post-stained in 5% uranyl acetate for 10 min and in Reynold's lead citrate for 5 min, sections were observed using a 40-120 kV transmission electron microscope (FEI TECNAI G2 Spirit Biotwin, Hong Kong, China). For the *in vitro* study, primary cardiomyocytes were collected by centrifuging at 1,000 rpm for 10 min and fixing them in 100 μL of 2% glutaraldehyde. The subsequent operations were performed as described above (Hu et al., 2017).

### Mitochondrial and Cytosolic Protein Extraction

Mitochondrial and cytosolic components were extracted using a Mitochondria Isolation Kit (C3601, C3606, Beyotime). First, cardiomyocytes were well-distributed by Mitochondria Isolation Solution containing PMSF in an ice bath for 15 min. A glass homogenizer was applied to grind the cells followed by centrifugation with 1,000 × g for 10 min at 4°C. The liquid supernatant was shifted to another tube and centrifuged again with 11,000 × g for 10 min. The sediment was blended with Mitochondrial Lysate Solution to obtain mitochondrial proteins. The supernatant was centrifuged with 12,000 × g for 20 min to obtain cytosolic proteins.

### Western Blot Analysis

After treatments, murine heart tissues and primary cardiomyocytes were collected, digested and analyzed using a BCA assay (for protein concentration, Thermo Fisher Scientific, USA). Protein samples of each group were separated using SDS-PAGE gels, transferred to the polyvinylidene difluoride membrane (PVDF, Millipore, USA), and then incubated overnight at 4 °C with specific antibodies against Mst1 (1:1,000, Cell Signaling Technology); t-Drp1 (1:1,000, Millipore, USA); p-Drp1^S616^ (1:500, Cell Signaling Technology, USA); p-Drp1^S637^ (1:500, Cell Signaling Technology, USA); Mff (1:1,000, Proteintech, USA); Mid49 (1:500, Proteintech, USA); Mid51 (1:1,000, Proteintech, USA); Fis1 (1:1,000, Proteintech, USA); Mfn1 (1:500, Proteintech, USA); Mfn2 (1:5,000, Abcam, USA); Opa1 (1:2,000, Abcam, USA); caspase-3 (1:1,000, Cell Signaling Technology, USA); cleaved caspase-3 (1:1,000, Cell Signaling Technology, USA) and β-actin (loading control, 1:1,000, Cell Signaling Technology). Then, blots were incubated with horseradish peroxidase (HSP)-conjugated secondary antibody (1:5,000, Proteintech, USA) for 1 h. Finally, blots were scanned and detected by the luminescence method. Band intensity was analyzed using the Image J software (Version 1.45).

### Real Time PCR

RNA was extracted with RNeasy mini kit (Qiagen, USA), cDNA was synthesized with a high-capacity cDNA reverse transcription kit (Applied Biosystems, Lithuania). Quantitative real-time PCR was performed using a Power SYBR Green PCR Master Mix (Applied Biosystems, UK). All procedures were performed strictly following the manufacturers' protocols. The primer sequences are as follows: Complex-IV forward CAGGATTCTTCTGAGCGTTCTATCA, Complex-IV reverse AATTCCTGTTGGAGGTCAGCA, NADH dehydrogenase subunit 1 forward ATGGTCAGTCTGTCATGGTGGAAC, NADH dehydrogenase subunit 1 reverse GCATAGCACAAGCAGCGACAAC, GAPDH forward ACGGCAAATTCAACGGCACAGTCA, GAPDH reverse TGGGGGCATCGGCAGAAGG.

### Immunofluorescence

For immunostaining, antibodies to Drp1 (1:500, Millipore, USA) and MitoTracker® Red CMXRos (40741ES50,) were used. Cells were grown in a particular vessel for fixed-cell imaging. After treatments, cells were washed twice with PBS and incubated with MitoTracker Red CMXRos for 20 min at 37°C. Cells were fixed in pre-warmed 4% paraformaldehyde for 10 min at 37°C, permeabilized in 0.1% Triton X-100, and all were washed after each step by phosphate-buffered saline. The primary antibodies were incubated for 48 h at 4°C and then conjugated for detection with Alexa fluor 488 anti-goat. The nuclei were dyed with DIPA. Scoring of mitochondrial morphology was performed blind to genotype in triplicates of 100 cells. Imaging was performed with a Plan-Apochromat 63 × /1.4 oil objective on a Zeiss LSM 710 confocal microscope driven by Zen 2009 software (Carl Zeiss, Jena, Germany). Images were cropped, globally adjusted for contrast and brightness, and median filtered using ImageJ (National Institutes of Health, Bethesda, MD).

### Adenovirus Construction and Transfection

The adenoviruses harboring Drp1 shRNA (Ad-sh-Drp1; MOI: 100); Mst1 shRNA (Ad-sh-Mst1; MOI: 100); Mst1 (Ad-Mst1; MOI: 100); and control vectors (Ad-sh-LacZ and Ad-LacZ; MOI: 100) were purchased from Hanbio Technology, Ltd. (Shanghai, China) and were transduced 4 h after the cardiomyocytes were treated with low (5.5 mM) or high (33 mM) glucose for 48 h.

### Determination of Mitochondrial Membrane Potential (MMP)

Primary cardiomyocytes were cultured in disposable confocal dishes. After their corresponding treatments, cells were rinsed with PBS and incubated with 5 μM JC-1 dye (C2006, Beyotime) at 37°C for 20 min. Fluorescent cells were visualized using an Olympus confocal microscope. Cellular mitochondria with normal MMP emitted red fluorescence (J-aggregate), while those with abnormal MMP showed green fluorescence (J-monomer). The mitochondrial membrane potential (MMP) was calculated using Image-Pro-Plus (Version 6.0) as red /green fluorescence.

### Mitochondria Biological Function

To assess the functional status of the mitochondria, an ATP bioluminescent assay kit (S0026, Beyotime) was used to detect the ATP level. The tissues were fully homogenized and centrifuged at 12,000 g for 15 min at 4°C. The supernatant was mixed with the corresponding reagent and assessed using a multimode microplate reader equipped with a luminescence luminometer (FLUOstar Omega, BMG Labtech, Germany). Citrate synthase and electron transport chain complex activities (complexes I/II/V) was measured using commercially available kits (Sigma, USA; Cayman, USA) according to the manufacturer's instructions. Data were representative of 4 biological repeats (Wang et al., 2018a).

### Statistical Analysis

All data were presented as the mean ± SEM. Differences between specific groups were determined by one-way analysis of variance (ANOVA) followed by Tukey's *post hoc* test (Graph-Pad 4.0, Graph Pad Software, La Jolla, CA, USA). Values with a *P* < 0.05 were considered statistically significant.

## Results

### Mst1 Inhibition Mitigates Cardiac Dysfunction and Cardiomyocyte Apoptosis in Diabetic Mice

**Figure 1:**
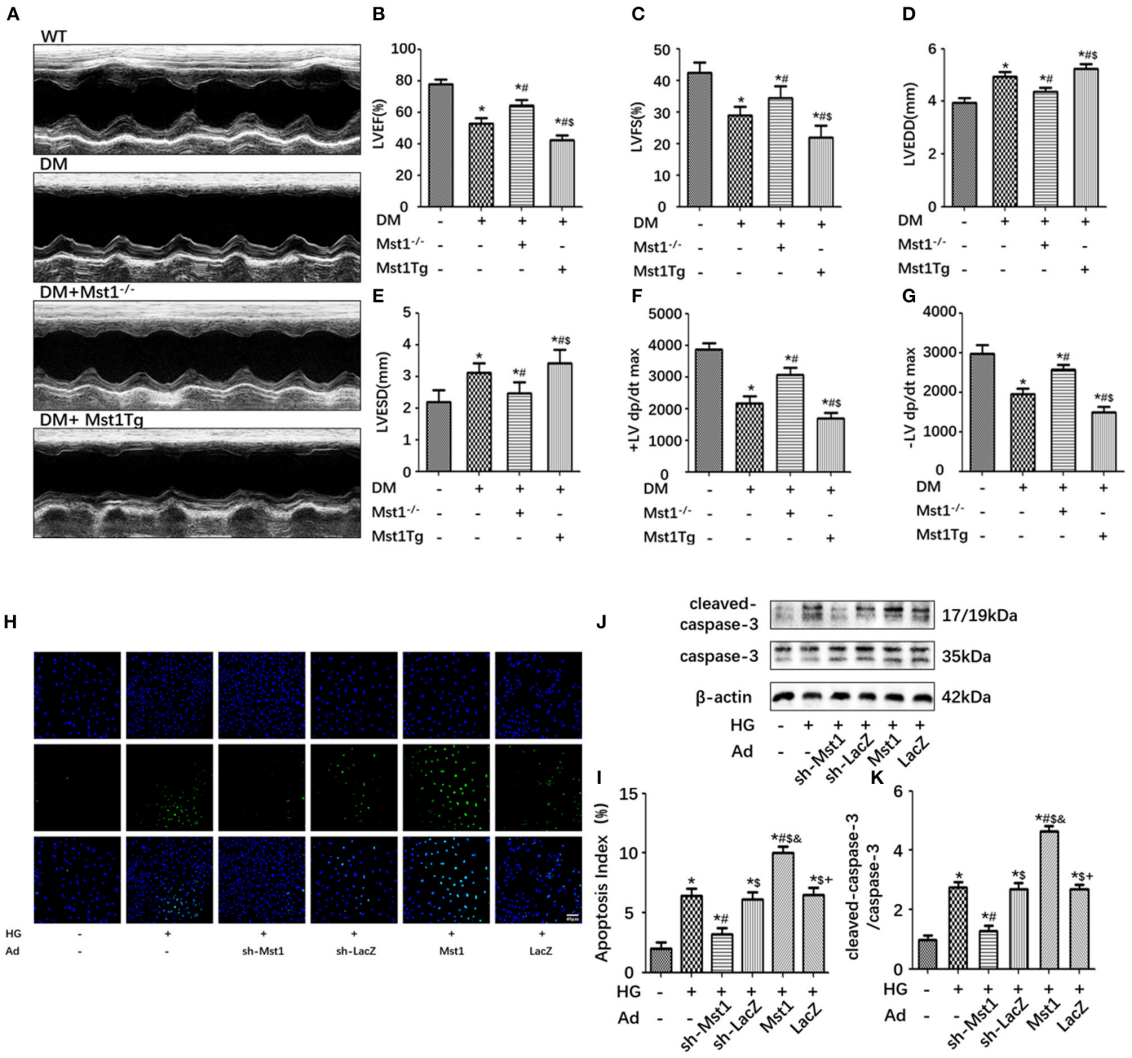
Mst1 inhibition alleviates cardiac dysfunction and cardiomyocyte apoptosis in diabetic mice. **(A)** Representative images of echocardiography. **(B)** Measurements of LVEF (%). **(C)** Measurements of LVFS (%). **(D)** Measurements of LVEDD (mm). **(E)** Measurements of LVESD (mm). **(F)** Hemodynamic evaluation of +LV dp/dt max. **(G)** Hemodynamic evaluation of -LV dp/dt max. The columns and error bars represent the means and SEM. *P < 0.05 vs. WT; ^#^*P* < 0.05 vs. DM; $*P* < 0.05 vs. DM + Mst1^−/−^. **(H)** Representative images of TUNEL staining in primary cardiomyocytes (scale bar: 40 mm). **(I)** Quantitative analysis of the apoptotic index (percentage of TUNEL-positive nuclei, %). **(J)** Representative western blots showing expression of cleaved-caspase 3, caspase 3 and β-actin (loading control) proteins. **(K)** Quantitative analysis of the ratio of cleaved-caspase 3/caspase 3. The columns and error bars represent the means and SEM. *P < 0.05 vs. Con; ^#^*P* < 0.05 vs. HG; $*P* < 0.05 vs. HG + Ad-sh-Mst1; and *P* < 0.05 vs. HG + Ad-sh-LacZ; and +*P* < 0.05 vs. HG + Ad-Mst1.

M-mode echocardiograms were used to quantify cardiac function. Representative M-mode echocardiograms were shown in Figure 1A. In diabetic mice, decreased LVEF and LVFS and increased LVESD and LVEDD were observed as compared with the WT mice. Mst1 overexpression inhibited, while Mst1 knockout enhanced LVEF and LVFS in mice underwent diabetes insult (Figures 1B,C). Mst1 knockout significantly inhibited left ventricular remodeling in diabetic mice, as evidenced by decreased LVESD and LVEDD (Figures 1D,E). Hemodynamic measurements also revealed that Mst1 knockout decreased ± LV dp/dt max and alleviated cardiac dysfunction in diabetic mice (Figures 1F,G).

Mst1 knockdown significantly decreased the number of TUNEL-positive cardiomyocytes subjected to high-glucose medium culture (Figures 1H,I). Furthermore, the ratio of cleaved caspase-3/caspase-3 was also reduced by Mst1 knockdown in cardiomyocytes cultured in high-glucose medium (Figures 1J,K). These data indicated that Mst1 deficiency ameliorated the cardiac pathological phenotype of diabetic mice.

### Mst1 Knockdown Ameliorates Mitochondrial Fission in the Experimental Diabetic Cardiomyopathy

**Figure 2:**
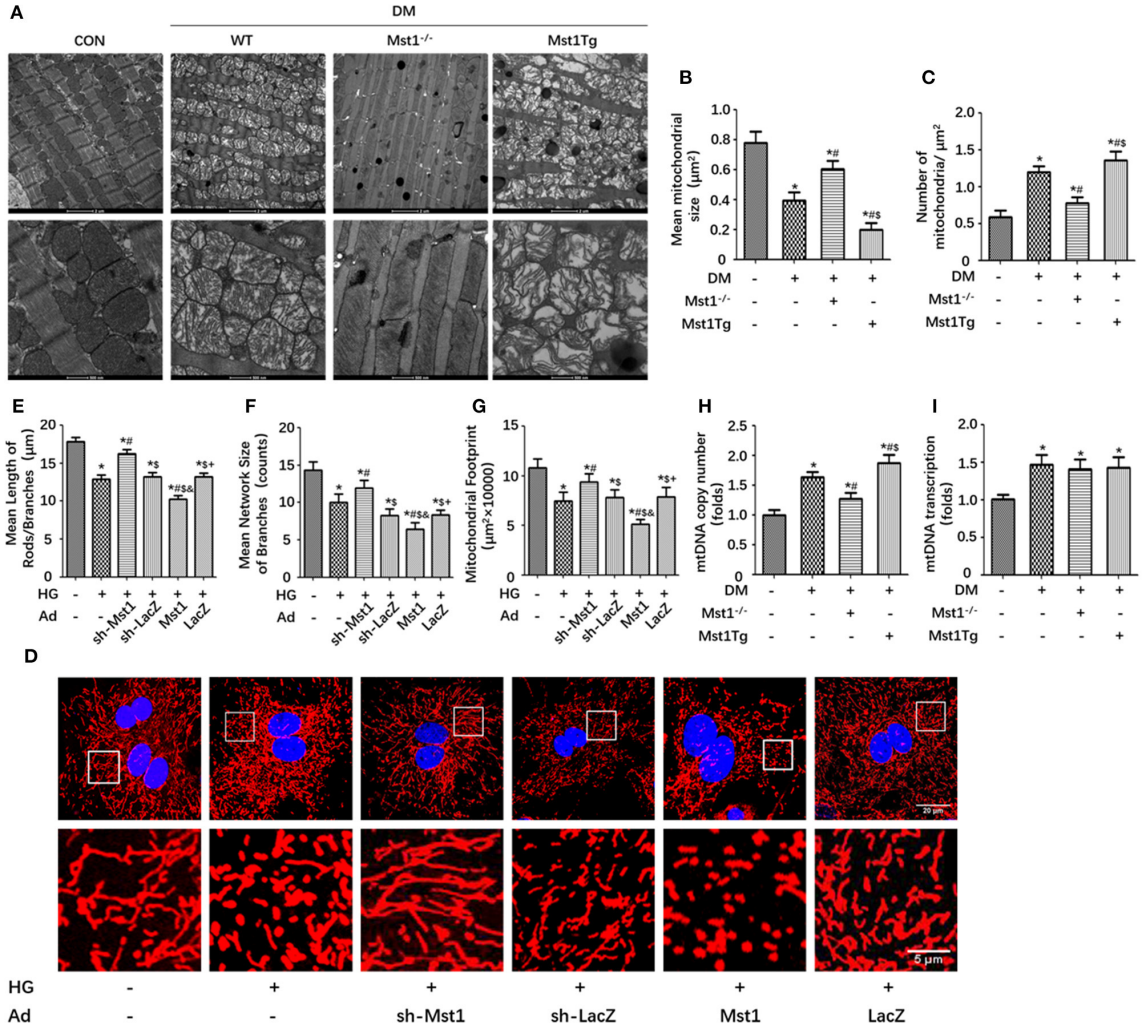
Mst1 participates in the regulation of mitochondrial dynamics. **(A)** Representative images of typical damaged and different sized mitochondria (Scale bars: upper panel 2 μm, lower panel 500 nm). **(B)** Quantitative analysis of the mean mitochondrial size. **(C)** Quantitative analysis of the number of mitochondria per μm^2^. The columns and error bars represent the means and SEM. *P < 0.05 vs. WT; ^#^*P* < 0.05 vs. DM; $*P* < 0.05 vs. DM +Mst1^−/−^. **(D)** Representative images of mitochondria (MitoTracker Red) (Scale bars: upper panel 20 μm, lower panel 5 μm). **(E)** Quantitative analysis of the mean length of mitochondria rods/branches. **(F)** Quantitative analysis of the number of branches. **(G)** Quantitative analysis of the mitochondrial footprint. The columns and error bars represent the means and SEM. *P < 0.05 vs. Con; ^#^*P* < 0.05 vs. HG; $*P* < 0.05 vs. HG + Ad-sh-Mst1; and *P* < 0.05 vs. HG + Ad-sh-LacZ; and +*P* < 0.05 vs. HG + Ad-Mst1. **(H)** mtDNA copy number was assessed by complex IV segment. **(I)** The transcript level of mtDNA was assessed by NADH dehydrogenase subunit 1 (ND1). The columns and error bars represent the means and SEM. *P < 0.05 vs. WT; ^#^*P* < 0.05 vs. DM; $*P* < 0.05 vs. DM +Mst1^−/−^.

The dynamic changes in mitochondrial fission and fusion were observed by transmission electron microscopy and confocal imaging. Transmission electron microscopy demonstrated that the mean mitochondrial size was lager, the number of mitochondria was decreased and mitochondrial crista damage was also ameliorated in the Mst1 knockout diabetic mice hearts, as compared with the diabetic mice (Figures 2A–C). Mst1 overexpression increased the prevalence of fragmented mitochondria, while Mst1 knockout induced a display of elongated mitochondria in the diabetic mice heart (Figures 2A–C). The Mitochondrial Network Analysis (MiNA) toolset, a simple ImageJ macro tool, classified mitochondrial structures as individuals and networks (Valente et al., 2017). MiNA could measure the average lengths of all rods/branches, evaluate the extent of mitochondrial branching and determine the total area in the image (Figures 2D–G). Mst1 overexpression decreased the length of rods/branches, the number of branches and the mitochondrial footprint, whereas Mst1 knockdown increased these parameters in cardiomyocytes subjected to high-glucose culture (Figures 2D–G). In addition, Mst1 knockout resulted in decreased mtDNA copy number as compared with the DM group. However, the transcript level of mtDNA was not significantly changed in Mst1 overexpression or knockout group (Figures 2H,I). Taken together, the above data indicated that Mst1 exacerbates mitochondrial fission and Mst1 knockdown ameliorates mitochondrial fission during DCM.

### Mst1 Knockout Decreases Drp1 Expression, Inhibits Drp1^S616^ Phosphorylation and Promotes Drp1^S637^ Phosphorylation in Diabetic Cardiomyopathy

**Figure 3:**
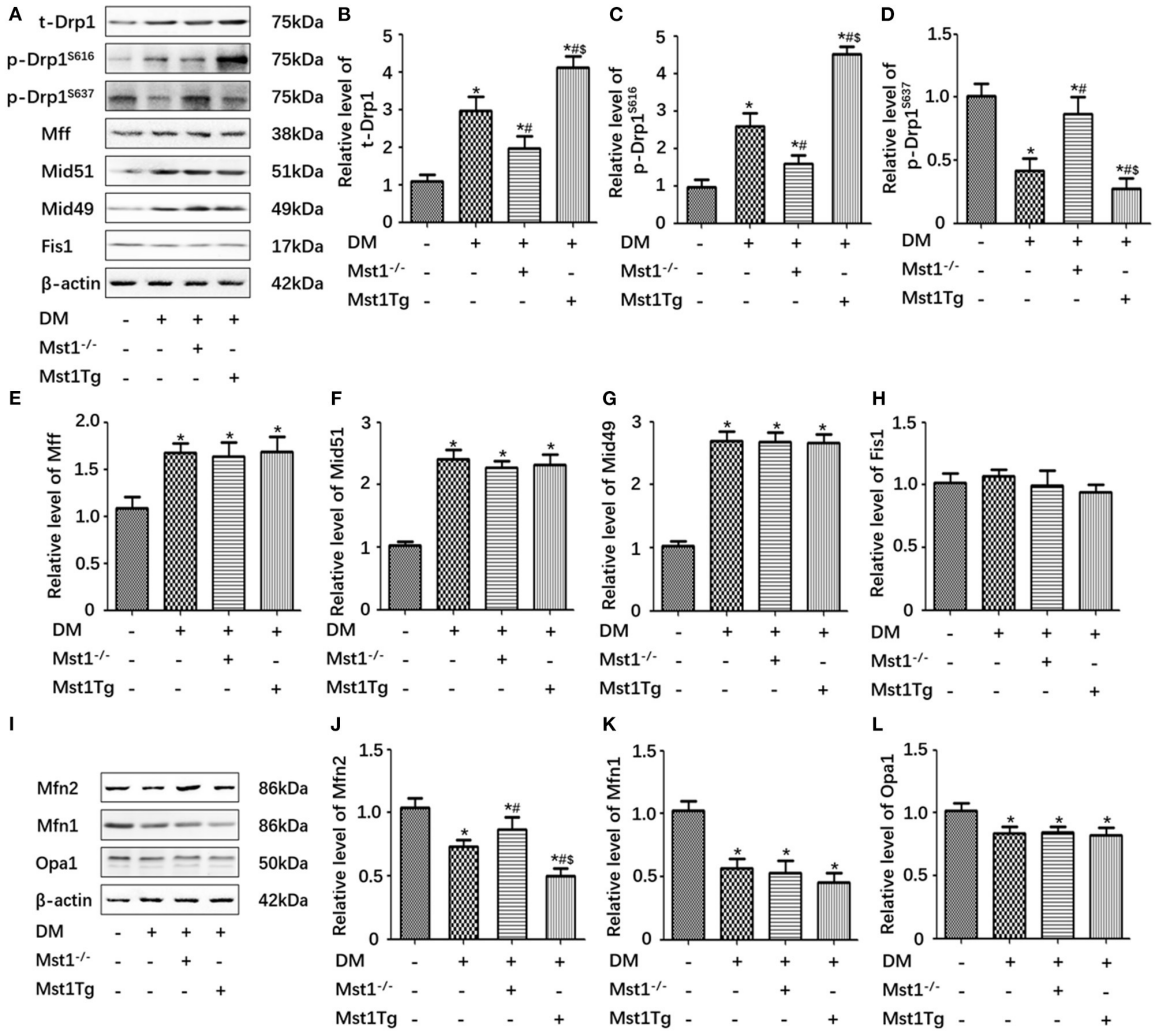
Mst1 knockout decreases Drp1 expression, inhibits Drp1^S616^ phosphorylation and promotes Drp1^S637^ phosphorylation in DCM. **(A)** Protein expression with representative gel blots of t-Drp1, p-Drp1^S616^, p-Drp1^S637^, Mff, Mid51, Mid49, Fis1, and β-actin (loading control for Drp1, Mff, Mid51, Mid49 and Fis1). **(B)** Relative level of t-Drp1. **(C)** Relative level of p-Drp1^S616^. **(D)** Relative level of p-Drp1^S637^. **(E)** Relative level of Mff. **(F)** Relative level of Mid51. **(G)** Relative level of Mid49. **(H)** Relative level of Fis1. **(I)** Protein expression with representative gel blots of Mfn2, Mfn1, Opa1, and β-actin (loading control for Mfn2, Mfn1, Opa1). **(J)** Relative level of Mfn2. **(K)** Relative level of Mfn1. **(L)** Relative level of Opa1. The columns and error bars represent the means and SEM. *P < 0.05 vs. WT; ^#^*P* < 0.05 vs. DM; and $*P* < 0.05 vs. HG +Mst1^−/−^.

Proteins of the mitochondrial fission machinery include Drp1, Mff, Mid49/51 and Fis1. MfnFN1, Mfn2 and Opa1 are involved consistently in mitochondrial fusion (Otera et al., 2013). Western blot analysis demonstrated that mitochondrial fission related proteins increased and mitochondrial fusion related proteins decreased in DM group compared with WT group (Figures 3A–L). Mst1 knockout decreased Drp1 expression, inhibited the phosphorylation of Drp1^S616^ and promoted the phosphorylation of Drp1^S637^. In contrast, Mst1Tg diabetic mice exhibited increased Drp1 expression, up-regulated phosphorylation of Drp1^S616^ and decreased phosphorylation of Drp1^S637^ (Figures 3A–D). Mst1 knockout did not significantly change the expression of MFF, Mid49/51 and FIS1 (Figures 3E–H). Furthermore, Mst1 knockout increased Mfn2 levels, while had no role on Mfn1 and Opa1 levels in DCM (Figures 3I–L).

### Drp1 Knockdown Abolishes the Effects of Mst1 on Mitochondrial Fission in Cardiomyocytes Subjected to High-Glucose Treatment

**Figure 4:**
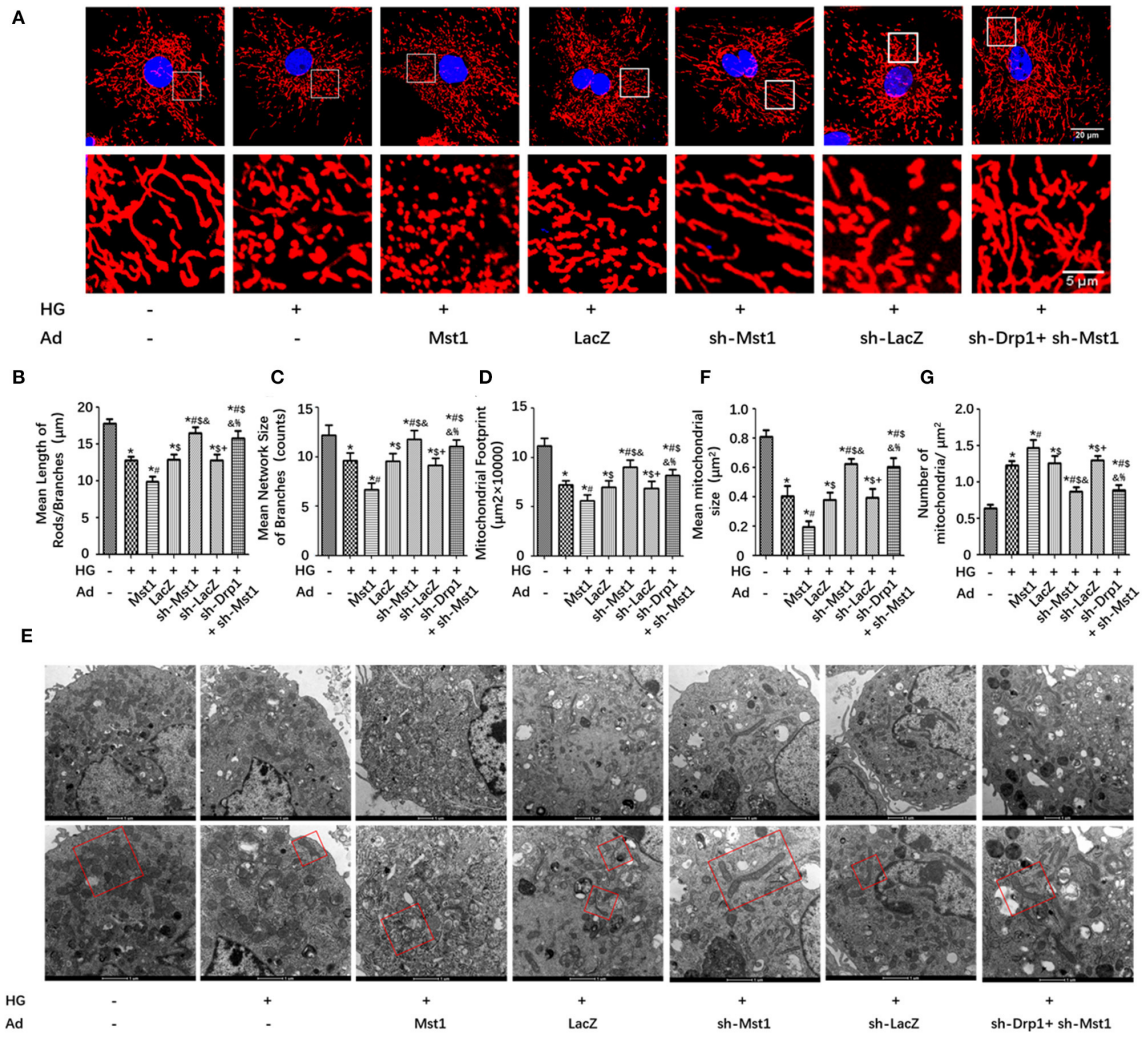
Drp1 knockdown abolishes the effects of Mst1 on mitochondrial fission. **(A)** Representative images of mitochondria (MitoTracker Red) (Scale bars: upper panel 20 μm, lower panel 5 μm). **(B)** Quantitative analysis of the mean length of mitochondria rods/branches. **(C)** Quantitative analysis of the number of branches. **(D)** Quantitative analysis of the mitochondrial footprint. **(E)** Representative images of typical damaged and different sized mitochondria (Scale bars: upper panel 1 μm, lower panel 500 nm). **(F)** Quantitative analysis of the mean mitochondrial size. **(G)** Quantitative analysis of the number of mitochondria per μm^2^. The columns and error bars represent the means and SEM. *P < 0.05 vs. Con; ^#^*P* < 0.05 vs. HG; $*P* < 0.05 vs. HG + Ad-Mst1; and *P* < 0.05 vs. HG + Ad-LacZ; +*P* < 0.05 vs. HG + Ad-sh- Mst1; and %*P* < 0.05 vs. HG + Ad-sh- LacZ.

In cardiomyocytes subjected to high-glucose culture, Mst1 knockdown significantly increased the length of mitochondrial rods/ branches, the number of branches and the mitochondrial footprint. Interestingly, Drp1 knockdown abolished the effects of Mst1 knockdown on the above parameters (Figures 4A–D). Consistently, Drp1 knockdown eliminated the role of Mst1 knockdown on the mean size of mitochondria and the number of mitochondria as evaluated by transmission electron microscopy (Figures 4E–G). These data indicated that Drp1 may serve as a downstream regulator of Mst1.

### Mst1 Knockdown Inhibits Drp1 Recruitment to the Mitochondria in Cardiomyocytes Under High-Glucose Treatment

**Figure 5:**
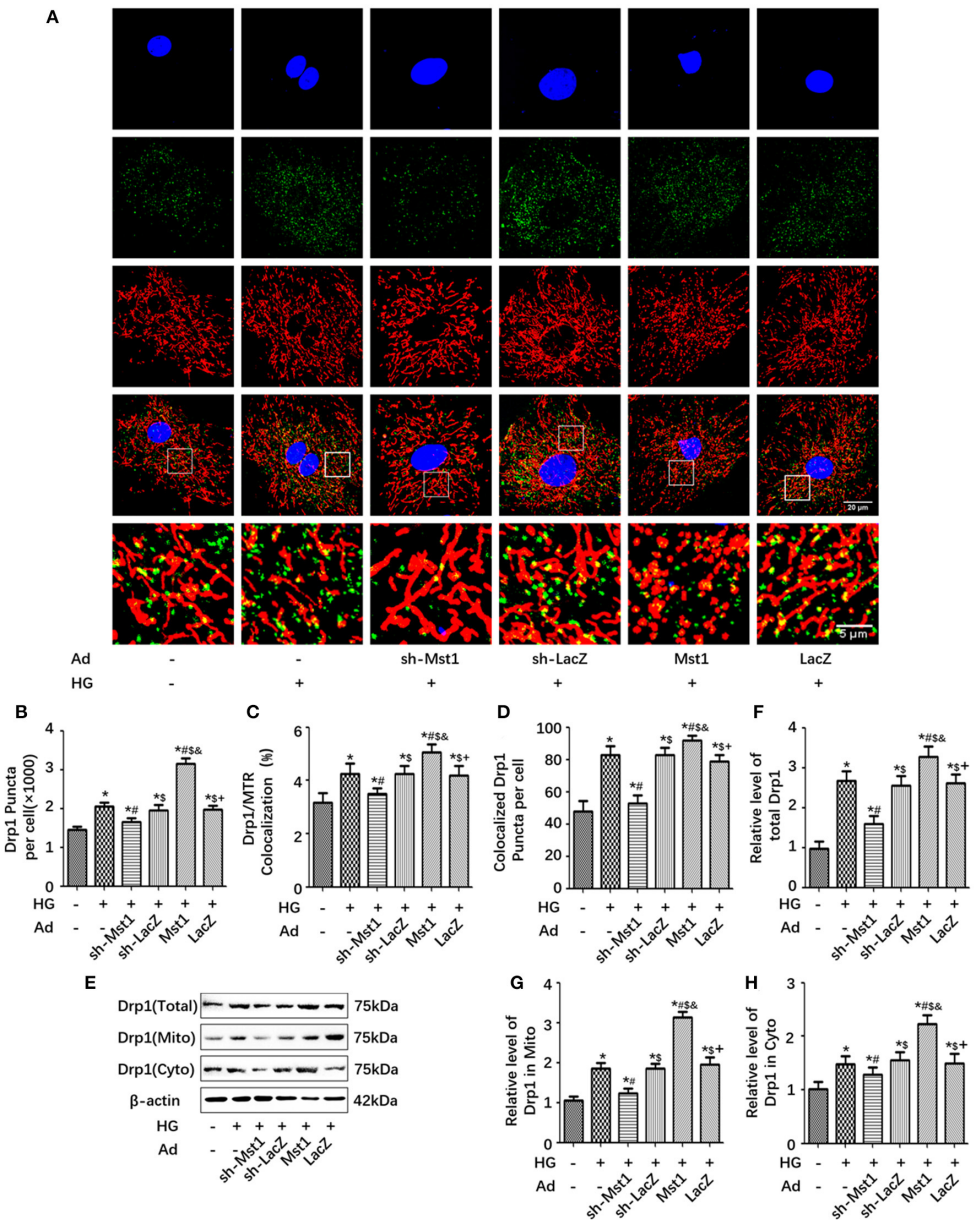
Mst1 knockdown inhibits Drp1 recruitment to the mitochondria. **(A)** Representative colocalization images of Drp1 (Green) and mitochondria (MitoTracker Red) (Scale bars: upper four panels 20 μm, lower panel 5 μm). **(B)** Quantitative analysis of Drp1 puncta per cell. **(C)** Quantitative analysis of Drp1/ MitoTracker Red colocalization. **(D)** Quantitative analysis of colocalized Drp1 Puncta per cell. **(E)** Protein expression with representative gel blots of total Drp1, mitochondrial Drp1 and cytoplasmic Drp1. **(F)** Relative level of total Drp1 **(G)** Relative level of Drp1 in mitochondria. **(H)** Relative level of Drp1 in cytoplasm. The columns and error bars represent the means and SEM. *P < 0.05 vs. Con; ^#^*P* < 0.05 vs. HG; $*P* < 0.05 vs. HG + Ad-sh-Mst1; and *P* < 0.05 vs. HG + Ad-sh-LacZ; and +*P* < 0.05 vs. HG + Ad-Mst1.

Drp1 is largely cytosolic with only 3% of the protein being associated with mitochondria (Smirnova et al., 2001). Drp1 transfer from the cytosol to the mitochondrion was essential for mitochondrial fission (Smirnova et al., 2001; van der Bliek et al., 2013). Immunofluorescence analysis was performed to observe the mitochondrial localization of Drp1. In cardiomyocyts subjected to high-glucose treatment, Drp1 was located increasingly on the mitochondria. Mst1 overexpression increased, while Mst1 knockdown decreased mitochondrial localization of Drp1 (Figures 5A–D). By isolating mitochondrial and cytosolic protein, Mst1 overexpression increased, while Mst1 knockdown reduced both the mitochondrial and cytosolic expression of Drp1 (Figures 5E–H). These results indicated that Mst1 triggers mitochondrial fission by promoting Drp1 expression and translocation to the mitochondria under HG treatment.

### Drp1 Knockdown Abolishes the Effects of Mst1 on Mitochondrial Membrane Potential and Mitochondrial Dysfunction

**Figure 6:**
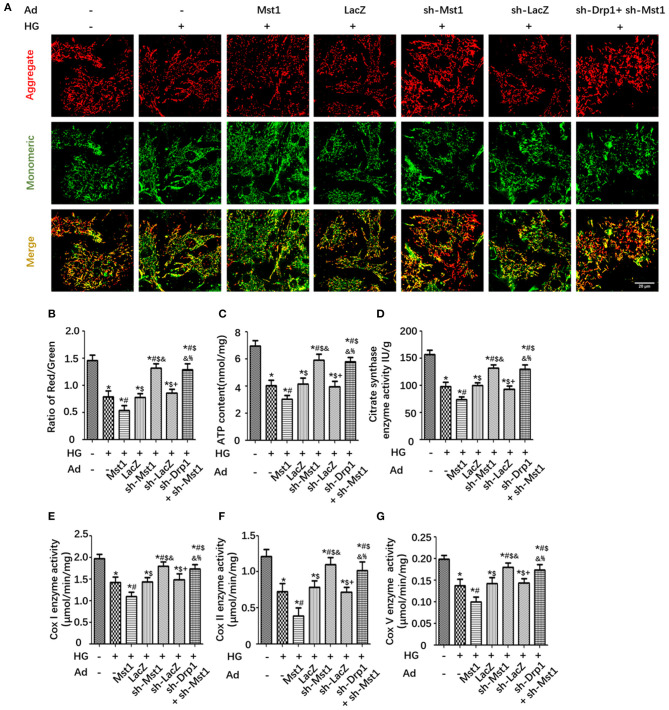
Drp1 knockdown abolishes the effects of Mst1 on mitochondrial membrane potential and mitochondrial dysfunction. **(A)** Representative images of JC-1 fluorescence imaging (Scale bar: 20 μm). **(B)** The ratio of aggregated (Red)/monomeric (Green) JC-1. **(C)** Cellular ATP content. **(D)** Citrate synthase (CS) activity. **(E)** Analysis of Complex I activity (ELISA assay) in primary cardiomyocytes. **(F)** Analysis of Complex II activity (ELISA assay) in primary cardiomyocytes. **(G)** Analysis of Complex V activity (ELISA assay) in primary cardiomyocytes. The columns and error bars represent the means and SEM. *P < 0.05 vs. Con; ^#^*P* < 0.05 vs. HG; $*P* < 0.05 vs. HG + Ad- Mst1; and *P* < 0.05 vs. HG + Ad-LacZ; +*P* < 0.05 vs. HG + Ad-sh- Mst1; and %*P* < 0.05 vs. HG + Ad-sh- LacZ.

Mst1 knockdown increased the mitochondrial membrane potential (ΔΨm) in HG treated cardiomyocytes as evidenced by JC-1 fluorescence imaging (Figures 6A,B). As expected, Drp1 knockdown abolished the effects of Mst1 knockdown on mitochondrial membrane potential (Figures 6A,B). Mst1 knockdown significantly enhanced mitochondrial ATP content and CS activity in cardiomyocytes underwent HG treatment. Interestingly, Mst1 knockdown did not further increase ATP content or CS activity in cardiomyocytes subjected to Drp1 knockdown (Figures 6C,D). Similar results were observed on mitochondrial complex I (Cox I), complex II (Cox II) and complex V (Cox V) enzyme activity (Figures 6E–G). These results indicated that Mst1 aggravates mitochondrial functional injury in diabetic hearts via Drp1.

## Discussion

Diabetes mellitus describes a group of metabolic disorders characterized by hyperglycemia due to the absolute or relative insufficiency of insulin production or actions caused by a combination of genetic and environmental factors (Alam et al., 2014; Harreiter and Roden, 2019). Diabetic patients have a higher risk of morbidity and mortality and more documented cases of cardiovascular complications than the general population (Henning, 2018). Diabetic cardiomyopathy (DCM) is manifested as abnormal cardiac structure and function in the absence of ischemic or hypertensive heart disease with diabetes that is due to cardiomyocytes being in a hyperglycemic environment for a long time (Vasquez-Trincado et al., 2016). However, the underlying molecular mechanism of diabetic cardiomyopathy remains unclear. Consistent with our previous study, the present project revealed that Mst1 aggravated the development of DCM (Zhang et al., 2016). Mst1 knockout significantly inhibited left ventricular remodeling, enhanced cardiac function, as well as decreased cardiomyocyte apoptosis in the diabetic heart.

Mitochondrial quality control includes post-translational modification (PTM) of mitochondrial proteins, mitochondrial dynamics (biogenesis, fission, and fusion) and mitochondrial autophagy (mitophagy) (Wu and Ren, 2006; Suliman and Piantadosi, 2016; Klimova et al., 2018; Pei et al., 2018). Our previous study demonstrated that Mst1 inhibits Sirt3 expression thus participates in the development of DCM by inhibiting cardiomyocyte mitophagy through inhibiting Parkin-dependent mitophagy (Wang et al., 2018a). Additionally, Mst1 has no effect on mitochondrial biogenesis, which was assessed by PGC-1α, NRF-1 and TFAM expression (Vega et al., 2015; Wang et al., 2018a). There is a large amount of evidence indicating that mitochondrial fission participates in cardiac metabolic disease (Mishra and Chan, 2016). Here, our work mainly demonstrates that Mst1 is involved in the regulation of mitochondrial fission, which is a crucial factor for exacerbating the mitochondrial dysfunction and cardiac remodeling of DCM mice.

Mitochondrial fission begins with the recruitment of cytoplasmic Drp1 by the adaptor on the mitochondrial membrane protein (MFF, Fis1, and Mid49/51) (Pagliuso et al., 2018). Accumulating evidence indicates that exposure of neonatal cardiomyocytes to sustained, high levels of glucose (35 mm) increased mitochondrial fission, reduced mitochondrial membrane potential (ΔΨm) and electron transport chain activity (Yu et al., 2011). Increased mitochondrial Drp1 translocation is a main mediator of mitochondrial fission and may serve as a therapeutic target against high glucose toxicity in DCM (Gawlowski et al., 2012). However, whether mitochondrial fission directly contributes to the cardiac dysfunction associated with diabetic cardiomyopathy is unknown. In the present study, we observed that Mst1 increased the expression and mitochondrial localization of Drp1. This finding indicates that Mst1 triggers mitochondrial fission by promoting Drp1 translocation to the mitochondria.

The effects of Drp1 on mitochondrial fission is regulated by the phosphorylation of two serine residues at the c-terminal guanosine triphosphatase (GTPase) effector domain of Drp1 (Fan et al., 2020). The phosphorylation of Drp1 at S616 activates mitochondrial fission. On the contrary, phosphorylation of Drp1 at S637 inhibits mitochondrial fission. Since Mst1 is a type of serine-threonine kinase comprising 487 amino acids. The present study thus investigated whether Mst1 regulated mitochondrial fission through Drp1 phosphorylation. Interestingly, Mst1 upregulated Drp1^S616^ phosphorylation, inhibited Drp1^S637^ phosphorylation, as well as promoting Drp1 recruitment to the mitochondria. Consistently, Drp1 knockdown eliminated the role of Mst1 knockdown on the mean size of mitochondria and the number of mitochondria as evaluated by transmission electron microscopy. There results further demonstrated that Mst1 is a crucial factor for exacerbating mitochondrial fission and dysfunction by coordinately mediates the phosphorylation of Drp1.

Mfn1 and Mfn2 are well-known mediators of the mitochondrial outer membrane fusion, while Opa1 mediates the fusion of the inner mitochondria membrane (Yang, 2015). It is possible to exchange the contents of two mitochondria so that the defective mitochondrion can regain the necessary components of the respiratory chain and mitochondrial DNA (Wai and Langer, 2016). The crossing and complementation of mitochondrial DNA molecules can also prevent the accumulation of mitochondrial DNA mutations (Chen et al., 2005). In the circumstances of emergency and hunger, mitochondria fuse to meet energy requirements, and the sharing of matrix metabolites maximizes the metabolic efficiency (Mishra and Chan, 2016). Mfn2 is a key protein in mitochondrial morphology (Chandhok et al., 2018; Yu et al., 2018). In the present study, Mst1 also decreased the expression of Mfn2. However, the concrete mechanism of Mfn2 regulation exerted by Mst1 still needs further investigation.

With the progression of diabetic cardiomyopathy, high glucose eventually induces cardiac hypertrophy, fibrosis, increased stiffness and cardiomyocyte loss (Tan et al., 2020). Our previous study indicated that downregulation of Mst1 alleviates cardiac fibrosis but has no effect on cardiac hypertrophy in db/db mice (Xiong et al., 2020). Mst1 serves as an important regulator of the cardiovascular system, playing a unique role in cardiomyocytes, cardiac stem cells/progenitor cells, macrophages, endothelial cells and fibroblasts (Wang et al., 2016; Cheng et al., 2018; Xiong et al., 2020). Mst1 signal pathways are involved in many cardiovascular diseases, including atherosclerosis, myocardial ischemic injury and cardiomyopathy (Yang et al., 2018). Interestingly, the new finding that Mst1 is involved in the regulation of mitochondrial dynamics suggests a possible mechanism in the pathological progression of DCM. Identifying such molecular pathways that control Drp1 alterations in DCM might recover the net balance of continual fission and fusion and provide a new direction for the diagnosis and treatment of DCM.

## Conclusion

We can conclude from this study that Mst1 aggravates mitochondrial fission, impairs mitochondrial energy metabolism and function, and aggravates cardiac dysfunction in DCM. Whether intervention in mitochondrial dynamics can reverse this damage might warrant further research efforts.

## Data Availability Statement

The original contributions presented in the study are included in the article/supplementary materials, further inquiries can be directed to the corresponding authors.

## Ethics Statement

The animal study was reviewed and approved by the Institutional Animal Care in the Xi'an International Medical Center.

## Author Contributions

DS, HW, and ZZ contributed to conception and design of the study, performed all the experiments, and wrote the manuscript. XF, SW, XY, JL, WM, YD, and YZ performed all cell and animal experiments and collected research data, contributed to analysis and interpretation of data. All authors read and approved the final manuscript.

## Conflict of Interest

The authors declare that the research was conducted in the absence of any commercial or financial relationships that could be construed as a potential conflict of interest.
